# Core-Shell Beads as Microreactors for Phylogrouping of *E. coli* Strains

**DOI:** 10.3390/mi11080761

**Published:** 2020-08-07

**Authors:** Lena Gorgannezhad, Kamalalayam Rajan Sreejith, Melody Christie, Jing Jin, Chin Hong Ooi, Mohammad Katouli, Helen Stratton, Nam-Trung Nguyen

**Affiliations:** 1 Queensland Micro- and Nanotechnology Centre, Nathan Campus, Griffith University, 170 Kessels Road, Brisbane, QLD 4111, Australia; lena.gorgannezhad@griffithuni.edu.au (L.G.); sreejith.kamalalayamrajan@griffithuni.edu.au (K.R.S.); jing.jin3@griffithuni.edu.au (J.J.); c.ooi@griffith.edu.au (C.H.O.); 2 School of Environment and Science, Nathan Campus, Griffith University, 170 Kessels Road, Brisbane, QLD 4111, Australia; m.christie@griffith.edu.au (M.C.); h.stratton@griffith.edu.au (H.S.); 3 Genecology Research Centre, School of Health and Sports Science, University of the Sunshine Coast, Maroochydore DC, Queensland 4558, Australia; mkatouli@usc.edu.au

**Keywords:** PCR, liquid marble, core-shell bead, phylogrouping, simultaneous detection

## Abstract

Multiplex polymerase chain reaction (PCR) is an effective tool for simultaneous detection of target genes. Nevertheless, their use has been restricted due to the intrinsic interference between primer pairs. Performing several single PCRs in an array format instead of a multiplex PCR is a simple way to overcome this obstacle. However, there are still major technical challenges in designing a new generation of single PCR microreactors with a small sample volume, rapid thermal cycling, and no evaporation during amplification. We report a simple and robust core-shell bead array for a series of single amplifications. Four core-shell beads with a polymer coating and PCR mixture were synthesized using liquid marble formation and subsequent photo polymerization. Each bead can detect one target gene. We constructed a customised system for thermal cycling of these core-shell beads. Phylogrouping of the *E. coli* strains was carried out based on the fluorescent signal of the core-shell beads. This platform can be a promising alternative for multiplex nucleic acid analyses due to its simplicity and high throughput. The platform reported here also reduces the cycling time and avoids evaporation as well as contamination of the sample during the amplification process.

## 1. Introduction

*Escherichia coli* (*E. coli*) is a bacterium that is frequently found in the environment and the intestine of humans and some animals. While most strains of *E. coli* are not harmful, some strains are pathogenic and can cause infectious diseases such as pneumonia, urinary tract infections, diarrhea, gastrointestinal, and extra-intestinal infections [1,2]. Phylogenetic studies of *E. coli* strains identified four main groups comprising A, B1, B2, and D [3,4]. The distribution within these groups is related to the host origin of the strains. Phylogroups B2 and D demonstrated to include virulent strains, expressing several virulence factors [5,6]. However, phylogroups A and B1 are the most predominant and commensal groups in human and animal [7,8,9].

Over the past few decades, a broad range of conventional genotyping methods were employed for the characterisation of *E. coli* phylogroups. These approaches can be divided into two main groups: (i) digestion-based methods such as restriction fragment length evaluation, pulsed-field gel electrophoresis (PFGE) [10]; and (ii) PCR-based methods including enterobacterial repetitive intergenic consensus sequence (ERIC)- PCR [11,12], randomly amplified polymorphic DNA (RAPD) analysis only [13] or coupled with biochemical fingerprinting [14], ribotyping analysis [15], and multiplex phylogrouping PCR [16,17]. The major bottlenecks of these conventional approaches are the time-consuming and elaborate laboratory procedures and the corresponding equipment. PFGE is a genotyping technique that is used for digesting bacterial nucleic acids by restriction enzymes followed by fragmented DNA analysis. This digestion may result in DNA patterns even with a very low number of bands. The ease of interpretation in PFGE makes it a handy approach for genotyping of bacterial isolates. Nevertheless, this method has a long processing time of 5 to 6 days as required for isolation and digestion of DNA before electrophoresis [18]. (ERIC)-PCR is a powerful, rapid, and profitable fingerprinting method to discriminate the different strains. However, the large number of protocol steps affects its repeatability and speed. Furthermore, this method needs gel electrophoresis as a mandatory step for identification [19,20]. RAPD is a popular genotyping approach. This method amplifies a large template of genomic DNA using short and arbitrary primers, generating highly polymorphic fragments profiles. These fragmented DNA can be used as microbial identification fingerprints. These profiles can be quickly analysed without PFGE [21], even though various normalization methods have been developed to solve the reproducibility problem of this method [22]. PCR ribotyping has also been introduced as a useful method for bacterial phylogrouping that relies on phylogenetic analyses of existing rRNA [23]. 

Multiplex (tri/tetra plex) is another common PCR-based molecular technique for *E. coli* phylogrouping. In this method, the specific primers are simultaneously used for the amplification of 4 genes whose presence/absence produces a genotype, which identifies an isolate as a member of one of 8 different phylogroups [4,24]. Among the above-mentioned approaches, PCR ribotyping and multiplex PCR have been adopted in many laboratories as the ideal approach for *E. coli* typing. However, there are still some obstacles that limit their application. For example, agarose gel-based DNA separation technique is a necessary step for both assays. Conventional agarose gel-based technique has limitations such as inter-laboratory difference in explaining banding patterns, time-consuming and complex processes, and the need for skilled personnel as well as expensive equipment [25,26]. Although some previously reported systems such as labelled probes have been also used for direct target gene identification without the need for electrophoresis, the implementation of these probe-based sensors is time consuming and requires multiple PCR product handling steps [27].

Microfluidics was successfully utilised for nucleic acid amplification [28]. Lab-on-a-chip systems attracted a great deal of attention from the research community and the industry due to their advantages such as real-time analysis, miniaturization, portability, and high throughput [29]. Microfluidic devices also have other distinct advantages such as providing multiplex experiments, consuming a small amount of samples, and integration of optical components in the same device [30,31,32,33,34]. Furthermore, droplet-based microfluidics allow for the precise control of sample volume with temperature control for actuation [35], sorting [36], and thermal cycling for amplification of nucleic acids. However, the major drawbacks of droplet-based microfluidics are complex fabrication processes, difficulties in sealing and valving, evaporation, and inconsistency. Furthermore, the design and fabrication of the corresponding devices need specialised instruments and expertise [37]. Thus, there is a need for novel real-time amplification methods for phylogrouping with features such as low cost, high speed, simple operation, and high sensitivity.

Liquid marble, a liquid droplet encapsulated by hydrophobic powder, can be an alternative platform to address the above issues of conventional methods. The powder coating prevents the direct contact between the core liquid and the surrounding, thus reducing contamination [38]. Liquid marble can maintain its stability on a solid surface, making handling a liquid becomes handling a solid [39]. The porous coating allows for introducing and removing reagents and reaction products into and from a liquid marble. Due to the small dimension of a liquid marble, the reagent consumption can be reduced [40,41], particularly as for chemical and biological applications [42]. More recently, Sreejith et al. [43] used liquid marbles as reactors for DNA amplification. Polytetrafluoroethylene (PTFE) was used as the hydrophobic coating. However, the overall thermal cycling time was limited due to the increasing rate of evaporation through the porous coating. The limited number of thermal cycles may negatively impact the amplification efficiency. The team employed a composite liquid marble as a bioreactor to reduce evaporation [44].

The present paper reports an amplification-based detection assay using four core shell beads for phylogrouping of *E. coli* strains. The PCR mixture was inserted into a photopolymer droplet, forming a spherical bead. The resulting liquid droplet was first embedded in a hydrophobic/oleophobic powder. The powder and the shell liquid not only provide the sterile condition for the PCR mixture, but also remarkably reduce evaporation. Moreover, solidification of the polymer transforms the liquid marble into a core-shell bead, enabling easy handling with no evaporation lost.

## 2. Materials and Methods

### 2.1. Fabrication of the Core-Shell Bead

**Figure 1:**
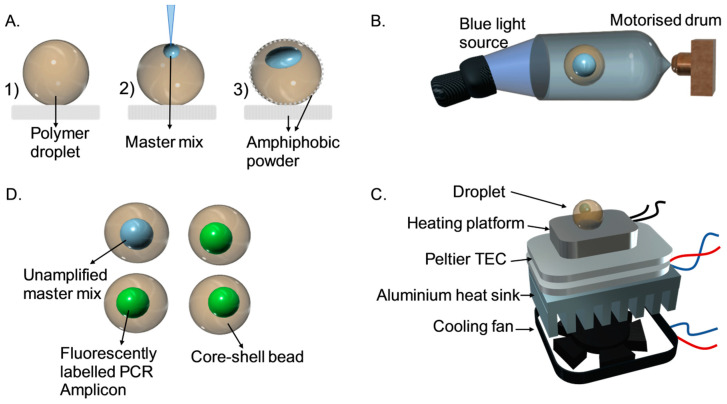
Operation of core-shell-bead-based polymerase chain reaction (PCR). (**A**) (1) Placing a droplet of photopolymer on top of an amphiphobic powder bed. (2) Inserting the PCR solution into the deposited droplet. (3) Covering the droplet with amphiphobic powder. (**B**) Photo polymerization of the droplet under blue light in a drum rotating at 140 rpm. (**C**) Transferring the generated core-shell bead to a custom-built thermal cycler for amplification. (**D**) Discriminating the core shell beads containing amplicons based on fluorescent intensity.

The synthesis and characterization of a core-shell bead was reported previously [44]. The synthesis of the bead consists of four main steps. First, a silicon-type gel monolith [45] was crushed and used as the super-amphiphobic powder bed. Next, 50 mg of camphorquinone (a photoinitiator) and 60 mg of 94 ethyl-4-(dimethylamino) benzoate were added to 10 g of trimethylolpropane trimethacrylate and mixed using a stirrer at 600 rpm for a few minutes. The prepared mixture was used as the photo polymer liquid. Subsequently, 20 µL of the photopolymer was deposited on the prepared powder bed. Next, 2 µL of the PCR solution was inserted into the photopolymer droplet. The composite droplet was then slowly rolled in the powder bed to create a protective amphiphobic coating (Figure 1A, steps 1–3). Finally, photopolymerization under blue light resulted in solidification of the polymer and hermetic encapsulation of the PCR master mix solution (Figure 1B). To centralize the injected PCR mixture inside the outer photopolymer droplet, photopolymerization was performed in a cylindrical drum rotating at 140 rpm for 5 min. Our results show that 5 min blue light illumination did not cause severe effect on photobleaching of the PCR products due to the short exposure time and the protective shell. However, our previous studies [28,44] indicated that constant exposure on the sample during thermal cycling over a long period of time can cause photobleaching of amplicons. This issue was solved by non-continuous illumination on the beads during thermal cycling. Lastly, the coating powder was removed using mineral oil, resulting in a transparent core-shell bead. The core shell bead is a solid bead, and no shape change was observed during the thermal cycling process.

### 2.2. Thermal Cycler

A custom-built thermal cycler was developed to provide the required conditions for PCR with the core shell beads [44]. A 2 cm × 2 cm × 1.5 cm aluminium block with an embedded cartridge heating element (0.5 cm diameter and 1.5 cm length, core-electronics) served as the heater. The aluminium block was attached to a Peltier thermoelectric cooler (4 cm × 4 cm × 0.35 cm, AUS-Electronics, NSW, Australia). An aluminium heat spreader and a cooling fan (12 V, 3,300 rpm, 7 cm × 7 cm × 2.5 cm) conduct the heat out of the setup, Figure 1C. For programable thermal cycling, a PID (proportional integral derivative) controller was implemented in an Arduino UNO board to provide the closed-loop control of the temperature. This thermal cycler generated the following temperature cycles: initial denaturation at 94 °C for 4 min, 30 cycles of denaturation at 94 °C for 5 sec each and annealing at 59 °C for 20 sec each, 5 min incubation at 72 °C, and infinite hold at 12 °C. More detail on the temperature profile during the PCR process and the control system were reported in our pervious study [44]. 

### 2.3. DNA Amplification

*E. coli* strains were isolated on nutrient agar plates from faeces samples in a clinical pathology lab. Single colonies were picked and grown overnight at 37 °C in nutrient broth. Isolates were stored at −80 °C until use in nutrient broth with 30% glycerol. The isolates were then used for phylogrouping experiment using single PCR and quadruplex PCR assays according to the method reported by Clermont et al. [46]. All samples were kept in ice before the experiment. After the sample was inserted into the bead, the sample stayed under room temperature of 23 °C for 5 Min before being transferred to the thermocycler. The experiments were carried out with one positive control (EC RBH2), three unknown samples, and one negative control (water). Table 1 lists the sequences of primers (forward and reverse) for the desired target genes. Single PCR utilised 20 µL of the PCR mixture containing SYBR Green, template DNA, one set of primer pairs, and water. The final concentration of the SYBR Green was 1×. The amounts of primer used were 20 pmol, except for AceK.f (40 pmol), ArpA1.r (40 pmol). Template DNA concentration was approximately 100 ng/µL. A volume of 2 µL of the prepared solution was inserted into the polymer droplet. After photopolymerization, the core-shell bead was placed on the customized thermal cycler. The samples underwent the aforementioned thermal cycling condition. For quadruplex PCR, the prepared PCR solution including all types of primer pairs, template DNA, and water was transferred to a conventional PCR instrument (Biorad CFX Connect, NSW, Australia) for thermal cycling. The final concentrations of the reagents were mentioned previously. The resulting amplicons were subsequently run on 2% agarose gel electrophoresis and followed by exposure of the UV transilluminator (Bio-Rad). *E. coli* strains were isolated by a clinical pathology lab in Turkey as part of the routine analyses of clinical samples. No clinical samples were directly collected from any patients, nor were the isolated strains part of any projects; therefore, there is no need for ethics approval for further testing them.

### 2.4. Optical Detection

**Figure 2:**
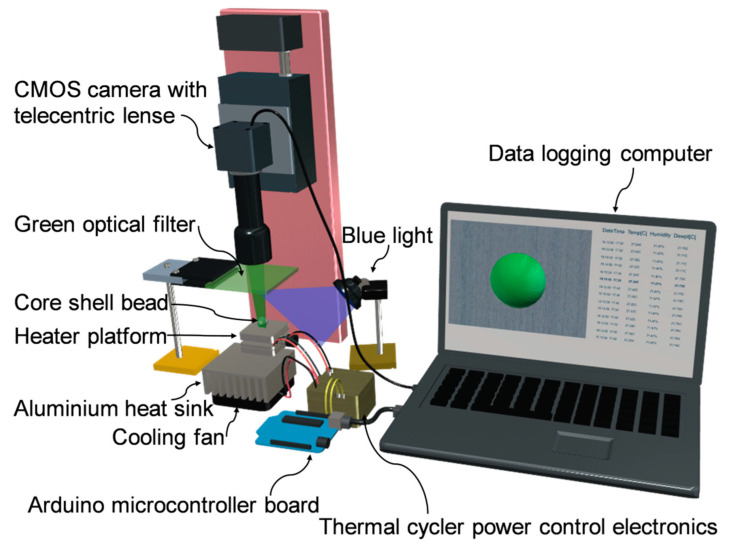
Schematic of the experimental setup.

For real-time analysis of the amplification, we developed a fluorescent detection platform as depicted in Figure 2. The range of the fluorescent excitation wavelengths was 450–490 nm (Blue light). The range of emission wavelengths was 520–560 nm (Green). A blue LED light (450–490nm) (1500mCd, Jaycar, Brisbane, Australia) served as the excitation source. Fluorescence measurement was performed at the end of the thermal cycles, and the fluorescent signal was recorded by a CMOS camera (EO-5012C, Edmund Optic, Singapore) equipped with a 0.5× telecentric lens (Edmund industrial Optics-63074). A green optical filter (520–560 nm) was also used for the emitted light to enhance the signal to noise ratio. The intensity of the emitted light provides information about the amplification potency.

## 3. Results and Discussion

### 3.1. Assay Principle

Using a core-shell bead as a microreactor (Figure 1), we demonstrate here an amplification-based assay for tracing marker genes and phylogrouping *E. coli* strains. For the phylogrouping experiment, four core-shell beads were fabricated. Each bead contained 2 µL PCR solution with a particular set of primer to detect one marker gene. The resulting core-shell beads were subsequently located on a customised PCR device for thermal cycling. A separator made of polydimethylsiloxane (PDMS) was used to prevent the beads from sticking to the heater during the thermal cycling process. The experiment was performed in a dark laboratory environment. The blue LED light source was employed to match the excitation wavelength. The light was manually turned on at five-cycle intervals. Experiments were repeated four times, one for positive control and three for unknown samples. Compared to the traditional phylogrouping method (PCR and subsequent gel electrophoresis), which take almost 3 h, our assay was carried out in around 1.5 h. One experiment with an unknown sample was repeated three times to test the reproducibility of the assay. Satisfactory results were achieved as shown in Figure S2. Moreover, based on our sensitivity measurements shown in Figure S3, the limit of detection (LOD) for the assay was 100 ng/µL. 

### 3.2. Phylogrouping Using Core Shell Beads

**Figure 3:**
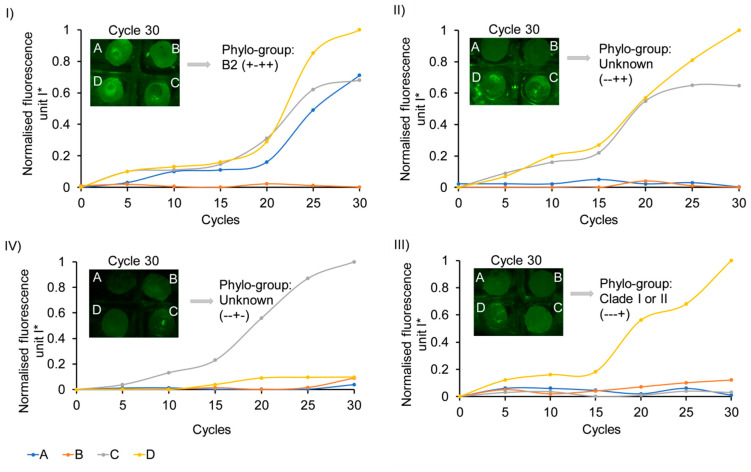
Amplification plot and phylogrouping of *E. coli* strains using core shell beads. (**I**) Positive control; (**II**–**IV**) unknown samples; A, B, C, and D beads containing specific set of primers to detect chuA, arpA, TspE4C2, yjaA genes individually; and images of core shell beads, are provided as insets.

We used core-shell beads of A, B, C, and D for the analysis of chuA, arpA, TspE4C2, and yjaA genes, respectively, to demonstrate the capability of our method for analysing clinical (control/unknown) samples. The numerical values of the fluorescent intensities of the amplicons inside the core shell beads were evaluated using ImageJ. The values were normalised as

(1) $$I_{\mathrm{sc}}^{*}=\left(I_{\mathrm{sc}}-I_{s0}\right)/I_{\max}$$

where $I_{\mathrm{sc}}$ is the fluorescence intensity of the sample measured in a given cycle, $I_{s0}$ is the fluorescence intensity of that sample at the beginning of the thermal cycling process, and $I_{\max}$ is the maximum fluorescent intensity measured between all samples. Figure S1 shows the representative images of the beads during an experiment to illustrate the increase in fluorescence intensity over the thermal cycling process. Figure 3 shows the amplification curves of the core-shell beads. Increasing the cycle number enhances the normalised fluorescence intensity of bead comprising target gene and related primer set, indicating a positive polymerase chain reaction within the bead. The lack of target gene inside a bead results in no significant fluorescence over thermal cycling. Each strain can be genotyped based on the presence/absence of the four genes in four beads. Figure 3 (I) shows phylogrouping of a positive control (EC RBH2). Beads A, C, and D show positive amplification. However, bead B has no significant amplification. The obtained genotype (+ − + +) indicates chuA +, arpA −, TspE4C2 +, and yjaA +, and subsequently an isolate is attributed to a phylo-group of B2. In addition to one positive control, three unknown samples were also evaluated using this assay. The corresponding phylo-groups are depicted in Table 2. Two of the *E. coli* isolates could not be exactly assigned to one of the known phylo-groups and reported as unknown strains. The multi-locus sequence type (MLST) technique [46] is required to assign these strain fractions.

### 3.3. Validation of Phylogrouping by Gel Electrophoresis

**Figure 4:**
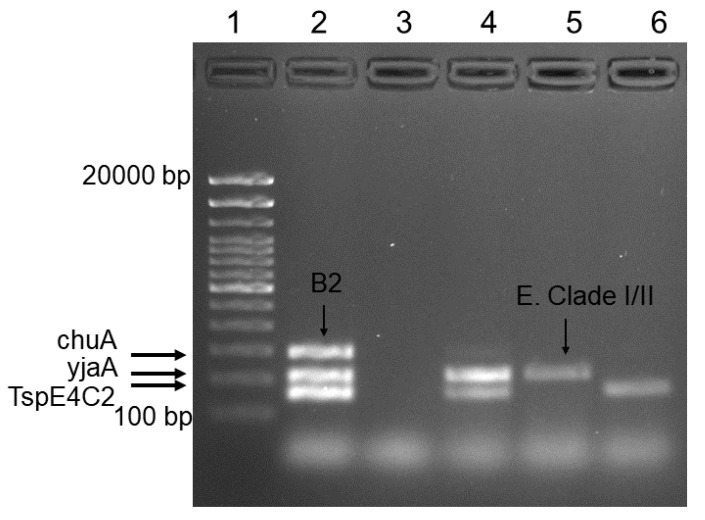
Agarose gel electrophoresis of amplicons from the Sybr-Green quadruplex PCR for confirmation. Lane 1: DNA ladder (100–20,000 bp). Lane 2: positive control (EC RBH2). Lane 3: negative control (water). Lane 4 to 6: unknown samples.

We used quadruplex PCR and gel electrophoresis to verify the phylogrouping results of the samples in a commercial platform. Figure 4 shows the result of Sybr-Green quadruplex PCR of positive control (EC RBH2), negative control (water), and unknown samples. Evaluation of the banding pattern on the gel showed three separate bands of 288 bp, 211 bp, and 152 bp for chuA, yjaA, and TspE4C2 genes, respectively. Lane 2 assigns to B2 strain of *E. coli* that possess chuA, yjaA, and TspE4C2 genes. *E.clade I/II* strain just shows amplification of yjaA gene (lane 5). The remaining genotypes (lane 4 and 6) cannot be classified into any distinct phylo-group. These results validate the accuracy of the amplification using core-shell beads.

## 4. Conclusions

Four core-shell polymer beads containing the PCR mixture were prepared by forming a liquid marble and subsequent photo polymerisation. A customised thermal cycler was developed to provide temperature control of the core-shell beads. Thermal cycling of the core-shell beads as PCR microreactor was performed for phylogrouping of the *E. coli* strains. This method potentially resolves the issues of conventional multiplex PCR in a single tube such as the intrinsic interference and the competition among primers, the requirement for optimized multiplex PCR protocols, and the difficulty in simultaneously detecting a large number of targets. Our assay demonstrated the following advantages: (i) simple fabrication and operation; (ii) fully sealed PCR microreactor that eliminates volume loss due to evaporation and minimises the risk of contamination; and (iii) reducing the PCR mixture volume to 2 µL, which decreases the cycle time. In summary, the core-shell bead platform reported here is capable of full functionality of a PCR assay due to its simplicity and potentially high throughput. This PCR microreactor can be employed in nucleic acid studies for clinical or environmental diagnostics. We believe that our method has a huge potential for commercialisation due to the reduction of expensive lab-based equipment and labour costs. Further development will focus on the automation of the platform, making it more user-friendly. Current manual fabrication of beads for a large number of samples is labour intensive. Thus, automation of bead preparation, partitioning of the PCR solution, and delivery into the fabricated beads can increase the speed and efficiency of the assay. Moreover, this platform has the potential to be fully integrated with other steps such as sample preparation, DNA extraction, amplification, and optical detection for the identification of bacteria as well as other basic research and translational applications.

## Figures and Tables

**Table 1:** Primer sequences and sizes of amplicons employed in the phylo-grouping experiment.

Target Gene	Primer ID	Sequence (5-3′)	PCR Product Size (bp)
**chuA**	chuA.1b chuA.2	ATGGTACCGGACGAACCAAC TGCCGCCAGTACCAAAGACA	288
**yjaA**	yjaA.1b yjaA.2b	CAAACGTGAAGTGTCAGGAG AATGCGTTCCTCAACCTGTG	211
**TspE4C2**	TspE4C2.1b TspE4C2.2b	CACTATTCGTAAGGTCATCC AGTTTATCGCTGCGGGTCGC	152
**arpA**	AceK.f ArpA1.r	AACGCTATTCGCCAGCTTGC TCTCCCCATACCGTACGCTA	400

**Table 2:** Genotypes of isolated *E. coli* samples.

Experiment ID	Bead A (chuA)	Bead B (arpA)	Bead C (TspE4C2)	Bead D (yjaA)	Phylo-Group
**I**	+	−	+	+	B2
**II**	−	−	+	+	Unknown
**III**	−	−	−	+	Clade I/II
**IV**	−	−	+	−	Unknown

## Supplementary Materials

The following are available online at https://www.mdpi.com/2072-666X/11/8/761/s1, Figure S1: Photographs of one set of the beads during thermal cycling, Figure S2: Reproducibility test of amplification for an unknown *E. coli* strain using core shell beads, Figure S3: Sensitivity test of amplification for one unknown *E. coli* strain with different concentrations using core shell beads.
