# Supplementary material for: Core-Shell Beads as Microreactors for Phylogrouping of E. coli Strains
Source: Micromachines (Basel). 2020 Aug 7;11(8):761. doi: 10.3390/mi11080761 (PMC7464145; doi:10.3390/mi11080761)
Supplement: Supplementary file 1 [file micromachines-11-00761-s001.pdf]

# Supplementary Materials: Core-Shell Beads as Microreactors for Phylogrouping of *E. coli* Strains

**Lena Gorgannezhad, Kamalalayam Rajan Sreejith, Melody Christie, Jing Jin,**

**Chin Hong Ooi, Mohammad Katouli, Helen Stratton and Nam-Trung Nguyen**

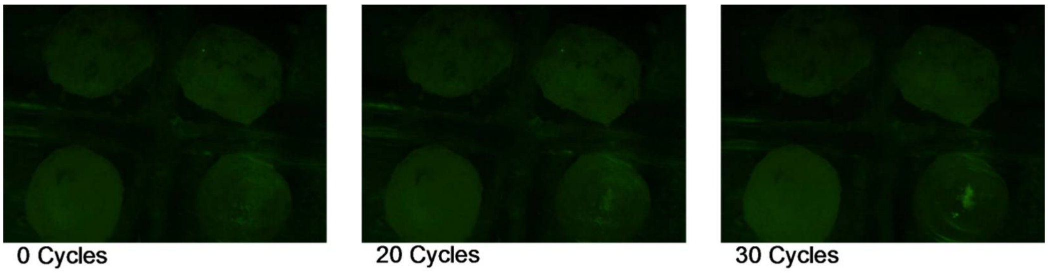


**Figure S1**. Photographs of one set of the beads during thermal cycling.

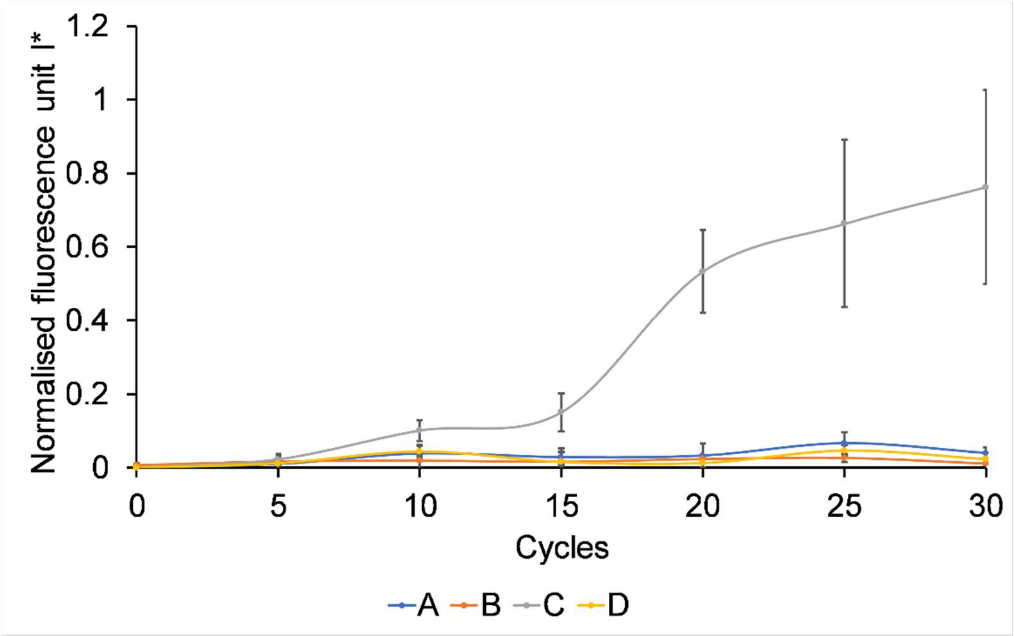


**Figure S2**. Reproducibility test of amplification for an unknown *E. coli* strain using core shell beads.

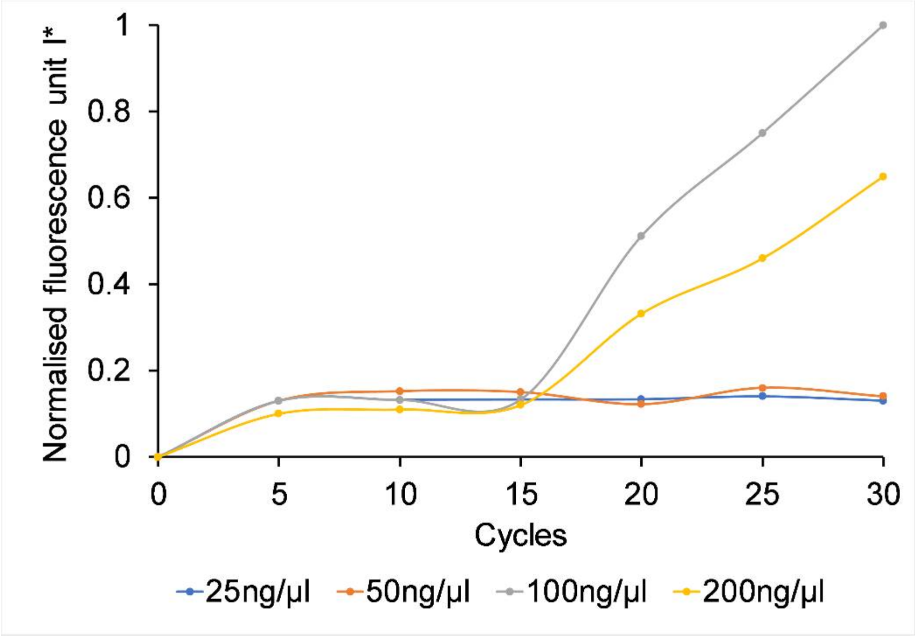


**Figure S3**. Sensitivity test of amplification for one unknown *E. coli* strain with different concentrations using core shell beads.
